# Supplementary material for: Patterns of Intron Gain and Loss in Fungi
Source: PLoS Biol. 2004 Nov 30;2(12):e422. doi: 10.1371/journal.pbio.0020422 (PMC532390; doi:10.1371/journal.pbio.0020422)
Supplement: Table S1 — Also available at http://genes.mit.edu/NielsenEtAl/. (4.3 MB ZIP). [file pbio.0020422.st001.zip › NielsenEtAl/html/186.html]

AN3778.1.NCU09746.1.MG06109.1.FG05299.1


```
 CLUSTAL W (1.82) Multiple Sequence Alignments - Introns Inserted


Sequence 1: NCU09746.1	725 aa
Sequence 2: MG06109.1	668 aa
Sequence 3: FG05299.1	672 aa
Sequence 4: AN3778.1	709 aa
Alignment Length: 764 aa
Number Identitical Residues: 326 aa
Alignment Score (without introns) 15855


MG06109.1 	MSK----QPLKAAILVVSTTAAHNPSTDATTAILQDVFDKDGAGQWTVVQAGIVTDSISD
NCU09746.1	MSSSTTPAALKAAILIVSTTAAQDPATDSADVTLRQVFDEQQA-QWEVHESKIVPDDVVQ
FG05299.1 	MAS----GPLKAALLIVSTTAAKDASADASGPILTQVLKDEGEGKWEVYDTKIVTDDVLA
AN3778.1  	MAE----QRLKAAILVVSDTAFQDPSSDRVFGTLIDVFPADGP--WEKPITKIVPDDVLQ
          	*:.      ****:*:** ** ::.::*     * :*:  :    *    : **.*.:  

MG06109.1 	IQRQVMSWTDTADAVN-------------LILTTGGTGFAVEDHTPE0AVGPLLHKQAPG
NCU09746.1	IQKQIIHWTNQATAASGDNNNNNNTNTINLIVTTGGTGFAVSDQTTE~AVSALLHKNAPG
FG05299.1 	IQRQITAWTDGSDSIN-------------LVVTTGGTGFATGDSTPE0AVTPLLHKQAPG
AN3778.1  	IQRAVCDWTDSPSPVN-------------LVLISGGTGFAVKDNTPE~AVTPLLHRHAPG
          	**: :  **: . . .             *:: :******. * *.* ** .***::***

MG06109.1 	LV~HGMIAASLNVTPF1AMMSRPVAGVRNGTIIVTLPGSPKGAKENLQAVIKLLPHACVQ
NCU09746.1	LV~HGMLAASLQVTPF~AMMSRPVAGVRNGTVIVALPGSPKGAKENLQAVIKMLPHACMQ
FG05299.1 	LV~HGMLAASLTVTPF1AMMSRPAAGVRNSTIIVTLPGSPKGAKENLQAIIKTLPHACIQ
AN3778.1  	LV2HGMIAASLKVTPF1AMMARPVAGTRHKSLVITLPGSPKGAKENLEAVIKLLPHACTQ
          	** ***:**** **** ***:**.**.*: :::::************:*:** ***** *

MG06109.1 	ASGANSRQLHSGGVEKLEQEAGVLAAESQSRGSSYHHHHHH-HHH---------------
NCU09746.1	AAGANSRALHKGGVKKLEQEAGVEGGGGRGHGHGHGHGHGH-KHHDHGEHRGGGGGGCGA
FG05299.1 	AAGANSRAIHSGGVKKLEAEAGISSKNKQPPATGHGCGHHH-HGHSHG------------
AN3778.1  	AAGADSRTLHAGGIKKLEAEAGVKSESQHDHHHHHHHEHTHSHSHSHG------------
          	*:**:** :* **::*** ***: .   :     :   * *:: *. .            

MG06109.1 	------------HGHDNLVRHTKPDSSPMSNDPNLGPTRRHRESPYPMLSVDEALNAIKT
NCU09746.1	HGHGHNHGHGPGHGHAGLVRHTIPSENPKSNDPSLGPSRRHRESPYPMLSVSEALSQIAQ
FG05299.1 	--------SGHGHSHGNLVRHTKPG-TTLSNDPALGPSRRYRESPYPMLPVKEALALIEQ
AN3778.1  	----------HGHGHVVPRAHTTPS-ERRSNDPAAGATRRYRESPYPMLSVDEALRQVSA
          	           .*.*     ** *.    ****  *.:**:********.*.***  :  

MG06109.1 	QTPTPTIETRKVNSGLVGAVLAEDVKAKENVPAFRASIVDGYAVVVPK---DGN-MRGVF
NCU09746.1	HTPMPQIVTSKVDSSLIGAVLAQDVSARENVPAFRASIVDGYAVVVPQ---DGN-MRGVF
FG05299.1 	HTPGPEVVTASVDQSIVNSVLAEDVEAKENVPAFRASIVDGYAVVAPK---DGQ-LKGVF
AN3778.1  	HTPEPEVIEVPVNIDLVGYVIAEDVYAAEAVPAYLASIVDGYAVIAPESPDDGHSTKGIF
          	:** * :    *: .::. *:*:** * * ***: *********:.*::..**:: :*:*

MG06109.1 	PVVSVSHASPGE---VPPLKEGEIARITTGAPLPPGATAVIMVEDTLLKTKT-------D
NCU09746.1	PVVSVSHAAPGDGEKMPTLKEGEIARITTGAPLPPGATSVIMVEDTVLVSTTSSKTEDAE
FG05299.1 	PVTAISHAAPGE---IGALKEGELARITTGAPLPPGATTVVMVEDTVLKTMS-------D
AN3778.1  	PVASITHANEEG--ALAPLEPGTIARITTGAPLPPNANAVVMVEDTLLASST-------P
          	**.:::**       : .*: * :***********.*.:*:*****:* : :        

MG06109.1 	DGN---EEKEVEVLAEG-VNDGENIREVGSDIERGTVVLRKYEQVSAAGGEIGSLAAVGV
NCU09746.1	DGKGVEEEKEVEILAADKVREGENVREVGSDIKQGQLILGKGDQVSAVGGEIGLLAAVGV
FG05299.1 	DEK---EEKEVEIQVEG-VKEGENIREVGSDVKQGTVILNKGETISGVGGEIGLLASVGV
AN3778.1  	DGK---EEATVEILTGE-IKPNENVRQPGSDVALGSRILQRGDLITPVGGEIGLLAATGT
          	* :   **  **: .   :. .**:*: ***:  *  :* : : :: .***** **:.*.

MG06109.1 	DEVQVFRRPTIAVLSTGDEIVQHDRPGELRLGEVRDTNRPTLIAAARHWGYEVIDMGIET
NCU09746.1	GEVQVYRRPVVGVLSTGDEIVDHDRPGDLRLGEVRDTNRPTLISAARDWGYEVIDLGVAR
FG05299.1 	SEAKVYRRPVVAVLSTGDEIVEYNRPGDLKLGEVRDTNRITLMSAAREWGYEVVDLGIAS
AN3778.1  	RTVKVFKKPVVGVLSTGDELVEHDDPRSLQGGQIRDSNRPSILSCLKSWGIPAVDLGIAR
          	  .:*:::*.:.*******:*::: * .*: *::**:** ::::. : **  .:*:*:  

MG06109.1 	DK-PGRLEETLRSALR-------QADVVITTGGVSMGELDLLKPTIERSLGGTIHFGRVS
NCU09746.1	DK-AGTLEETLRQALR-------QVDVVITTGGVSMGELDLLKPTIERSLGGTIRFGRVA
FG05299.1 	DK-PGTLEETLRDGLR-------RADLLITTGGVSMGELDLLKPTIERSLGGTIHFGRVA
AN3778.1  	DTPAGELEQSLRDALRGVGKSNTSVDVIITTGGVSMGELDLLKPTIERSLGGTIHFGRVS
          	*...* **::**..**. ..:.: .*::**************************:****:

MG06109.1 	MKPGKPTTFATVPVKDNS-----GERAQKVIFSLPGNPASALVTFHIFVLPSLHRLSGLQ
NCU09746.1	MKPGKPTTFATVPVKDND-----GERVSKVIFSLPGNPASALVTFHLFVLPSLHQMSGVS
FG05299.1 	MKPGKPTTFATVPVKDNA-----GQRVIKAIFSLPGNPASALVTFHLFVLPSLHKLSGAS
AN3778.1  	MKPGKPTTFATVPFKPTSSAAGQQERSSRLIFSLPGNPASALVTLNLFVLPSLHKLIGLG
          	*************.* . :::.. :*  : **************:::*******:: *  

MG06109.1 	----------AVGLPKIPVTLAHDFPLDKTRPEYHRAVVSVG-ADGLLSANSTG----GQ
NCU09746.1	----------PPGLPKVPVTLGHDFSLDRSRPEYHRAIVSVS-RDGVLTAVSTG----GQ
FG05299.1 	----------TVGLPRVPAVVSHDFPLD-PRPEYHRAVVTVG-KDGLLSATSTG----GQ
AN3778.1  	QKQAALGIAPALGLPLVAVTLSHAFPLDPKRTEYHRAIVTASPKDGRLYATSTGAEGVGQ
          	...:: . :.. *** :...:.* *.**  *.*****:*:... ** * * ***:.. **

MG06109.1 	RSSKVGSLKGANALLCLPNGAGPMKKGEQAVALLMGAIHGNN-----
NCU09746.1	RSSRVGSMKGANALLCMPCGPEPLRKGAKVEALLMGGLRSDV-----
FG05299.1 	RSSKVGNMRSANALVILPSGKGKLEKGSKVEVLLLNAVQAA------
AN3778.1  	RSSRVGSLASANSLLVLQPGKGSIAQGSLVEALMMGPIVREGAAVAL
          	***:**.: .**:*: :  *   : :*  . .*::. :    :: :
```
